# Supplementary material for: Comparative Analysis of SARS-CoV-2 Antibody Responses across Global and Lesser-Studied Vaccines
Source: Vaccines (Basel). 2024 Mar 19;12(3):326. doi: 10.3390/vaccines12030326 (PMC10975599; doi:10.3390/vaccines12030326)
Supplement: Supplementary file 1 [file vaccines-12-00326-s001.zip › vaccines-2850506-supplementary.pdf]

Methods

ELISA Procedure

Strips of 8 wells (Masixorp, loose nunc module 469949, Thermo Scientific) were coated overnight at 4°Celsius with 50 ul per well of a 2 ug/ml solution of S protein suspended in Phosphate Buffer Salina (PBS, Gibco, cat 14190-136) overnight. Subsequently, the coating solution was removed, and 100 ul per well of 4 % bovine serum albumin (B.S.A., Fisher BP1605-100, fraction V) prepared in PBS with 0.1% Tween 20 (TPBS) as blocking solution, were added and incubated at 37°C for 30 minutes. Serum samples were heated at 56°C for 1 hour before use to reduce the risk of any potential residual virus in serum. Serial dilutions on base 10 from 1:100 until 1;100000 serum samples were prepared in 5% non-fat milk plus 2.5% diluted in TPBS. Four negative controls and two positive controls were included in each run, The blocking solution was removed, and 100 ul of each serial dilution was added to the strips for 1 hour at 37ºC. Next, the strips were washed five times with 250 ul per well of TPBS. Next, a 1:10000 dilution of goat anti-human IgG-horseradish peroxidase-conjugated secondary antibody (anti-human IgG, gamma chain specific, SIGMA cat: A8419) in TPBS and 100 ul of this secondary antibody was added to each well for 30 minutes. Strips were again washed 250 ul seven times with 0.1% TPBS. One hundred ul of TMB (SIGMA cat: T0440) solution was added to each well. This substrate was left on the strips for 10 minutes, and then the reaction was stopped by adding 50 uL per well of 12.5% hydrochloric acid (HCl). The optical density at 450 nanometers with 560 nanometers of differential was measured in an ELISA reader (Thermo Fisher, model 5111340).

The cut-off for the discrimination of a seropositive signal was calculated as media negative controls multiplied by 3.8. The geometric mean titer (GMT) was calculated for each serum sample using an adaption of the Reed and Muench method as follows:

Titer= anti log 10 (OD last dilution above the cut-off – cut-off)/ (OD last dilution above the cut-off – OD first dilution<cut-off)) + log in base 10 from last dilution>cut-off

$$\mathrm{GMT} = 10^{[((OD_{last\ dilution>CO} - CO)/(OD_{last\ dilution>CO} - (OD_{last\ dilution<CO})) + log10_{last\ dilution>CO}]}$$

Survey Methods

Survey data was collected on paper using Remark Office O.M.R. forms. Information was scanned and extracted automatically into a database with multiple quality control validation steps.

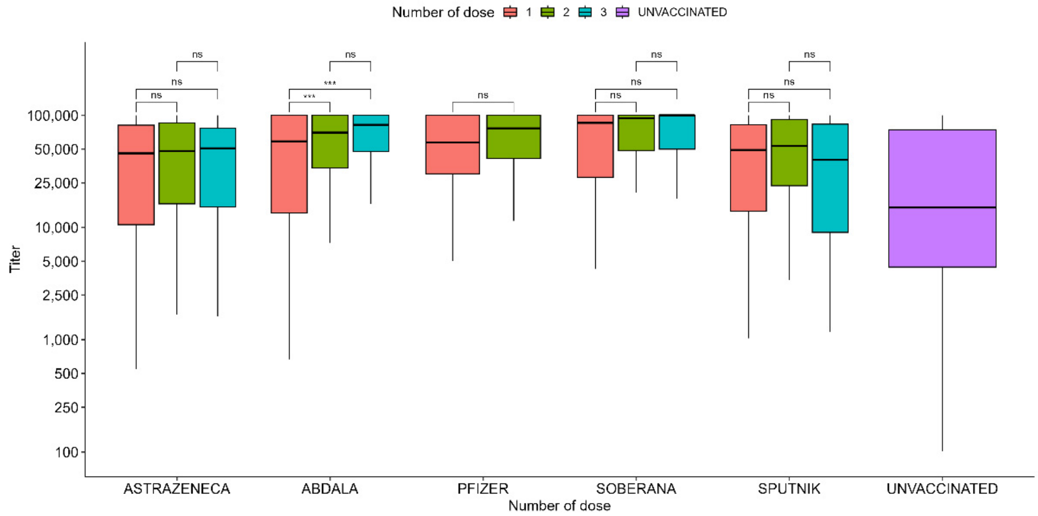

Figure S1. Anti-Spike antibody titers per vaccine and number of dose. Boxplots showing the anti-spike antibody magnitude by number of dose. T-test p-values from pairwise comparisons are shown

above boxplots. Significant p-values are shown with stars. N.S is a p-value > 0.05, one star is a p-value less than 0.05, two stars are a p-value less than 0.01, and three stars are a p-value less than 0.001.

Table S1. Participant demographics, vaccination status, number of doses, self-reported COVID and anti-spike antibody titer distribution.

	Overall	AstraZeneca	Abdala	Pfizer	Soberana	Sputnik	Unvaccinated
**N**	3195	802	801	272	270	717	333
**Vaccinated = Yes (%)**	2862 (89.6)	802 (100.0)	801 (100.0)	272 (100.0)	270 (100.0)	717 (100.0)	-
**Health Center (%)**							
**Roger Osorio**	955 (29.9)	235 (29.3)	240 (30.0)	81 (29.8)	80 (29.6)	211 (29.4)	108 (32.4)
**Silvia Ferrufino**	910 (28.5)	229 (28.6)	239 (29.8)	88 (32.4)	75 (27.8)	194 (27.1)	85 (25.5)
**Villa Libertad**	1330 (41.6)	338 (42.1)	322 (40.2)	103 (37.9)	115 (42.6)	312 (43.5)	140 (42.0)
**Age group (%)**							
**2-11**	590 (18.4)	0 (0.0)	248 (30.8)	0 (0.0)	267 (98.9)	0 (0.0)	75 (22.5)
**12-17**	631 (19.7)	0 (0.0)	547 (68.0)	14 (5.1)	3 (1.1)	0 (0.0)	67 (20.1)
**18-29**	632 (19.7)	192 (23.9)	10 (1.2)	158 (57.9)	0 (0.0)	204 (28.5)	68 (20.4)
**30-59**	726 (22.7)	308 (38.4)	0 (0.0)	101 (37.0)	0 (0.0)	246 (34.3)	71 (21.3)
**>=60**	622 (19.4)	303 (37.7)	0 (0.0)	0 (0.0)	0 (0.0)	267 (37.2)	52 (15.6)
**Sex = Female (%)**	2067 (64.7)	535 (66.7)	454 (56.7)	267 (98.2)	135 (50.0)	472 (65.8)	204 (61.3)
**Vaccination status (%)**							
**Incomplete**	1405 (49.1)	367 (45.8)	575 (71.8)	138 (50.7)	192 (71.1)	133 (18.5)	-
**Complete**	1027 (35.9)	239 (29.8)	226 (28.2)	134 (49.3)	78 (28.9)	350 (48.8)	-
**Heterotypic boost**	43 (1.5)	39 (4.9)	0 (0.0)	0 (0.0)	0 (0.0)	4 (0.6)	-
**Homotypic boost**	387 (13.5)	157 (19.6)	0 (0.0)	0 (0.0)	0 (0.0)	230 (32.1)	-
**Days after last dose (median (Q1-Q3))**	93 (39 - 206)	104 (53 - 246)	68 (33 - 146)	92 (35 - 161)	55 (23 - 120)	120 (55 - 234)	-
**Number of dose (%)**							
**1**	1334 (46.6)	367 (45.8)	296 (37.0)	137 (50.4)	101 (37.4)	433 (60.4)	-
**2**	932 (32.6)	240 (29.9)	279 (34.8)	135 (49.6)	91 (33.7)	187 (26.1)	-
**3**	596 (20.8)	195 (24.3)	226 (28.2)	0 (0.0)	78 (28.9)	97 (13.5)	-
**COVID (Self-reported) (%)**							
**Yes**	416 (13.0)	154 (19.2)	37 (4.6)	35 (12.9)	5 (1.9)	147 (20.5)	38 (11.4)
**Unknown**	109 (3.4)	33 (4.1)	16 (2.0)	8 (2.9)	5 (1.9)	28 (3.9)	19 (5.7)
**No**	2669 (83.6)	615 (76.7)	747 (93.4)	229 (84.2)	260 (96.3)	542 (75.6)	276 (82.9)
**Titer (median (Q1-Q3))**	56692 (14080 - 97883)	47764 (12860-82321)	67501 (28480-100001)	67314 (35454-100001)	94062 (41688-100001)	49048 (14091-85325)	10867 (3330-70641)
**Titer group (%)**							
**<100**	37 (1.2)	3 (0.4)	14 (1.7)	1 (0.4)	1 (0.4)	2 (0.3)	16 (4.8)
**100-1000**	116 (3.6)	19 (2.4)	36 (4.5)	5 (1.8)	4 (1.5)	14 (2.0)	38 (11.4)
**1001-5000**	173 (5.4)	51 (6.4)	20 (2.5)	8 (2.9)	8 (3.0)	36 (5.0)	50 (15.0)
**5001-10000**	385 (12.1)	109 (13.6)	66 (8.2)	21 (7.7)	23 (8.5)	106 (14.8)	60 (18.0)
**10001-50000**	764 (23.9)	228 (28.4)	161 (20.1)	68 (25.0)	43 (15.9)	202 (28.2)	62 (18.6)
**50001-100001**	948 (29.7)	250 (31.2)	246 (30.7)	87 (32.0)	61 (22.6)	237 (33.1)	67 (20.1)
**>100000**	772 (24.2)	142 (17.7)	258 (32.2)	82 (30.1)	130 (48.1)	120 (16.7)	40 (12.0)

Table S2. Seroprevalence of N antibodies in a random subset of unvaccinated participants by age, sex and health center population.

Variable	Categories	N	N seroprevalence (%)	Fisher test
**Overall**	-	100	81 (71.67 - 87.89)	-
**Age group**	2-11	20	75 (50.59 - 90.41)	0.8516
	12-17	19	89.47 (65.46 - 98.16)	
	18-29	19	78.95 (53.9 - 93.03)	
	30-59	29	79.31 (59.74 - 91.29)	
	>60	13	84.62 (53.66 - 97.29)	
**Sex**	Female	77	80.52 (69.6 - 88.34)	1.000
	Male	23	82.61 (60.45 - 94.28)	
**Health Center**	Roger Osorio	29	82.76 (63.51 - 93.47)	0.8014
	Silvia Ferrufino	25	76 (54.48 - 89.84)	
	Villa Libertad	46	82.61 (68.05 - 91.68)	

Table S3. Antibody titer magnitude difference among vaccinated vs. unvaccinated individuals.

				Univariate Analysis		Multivariate Analysis	
Variable	Category	n	Median titer	Model estimate *	pvalue	Model estimate **	p-value
Vaccinated	No	333	10867	Ref.	-	Ref.	-
	Yes	2862	59774	**22303.5 [18155.6, 26451.5]**	**<0.001**	**22897.5 [18779, 27016]**	**<0.001**
Age groups	2-11 y/o	622	71494	10452.5 [6294.2, 14610.8]	<0.001	**11448.9 [7361.4, 15536.5]**	**<0.001**
	12-17 y/o	590	65560	**8417.3 [4322.4, 12512.1]**	**<0.001**	**8949.8 [4927.4, 12972.2]**	**<0.001**
	18-29 y/o	627	52345	**1841.6 [-2246.8, 5929.9]**	**0.38**	2394.9 [-1621.3, 6411]	0.24
	30-59 y/o	631	47262	-855.4 [-4810.0, 3099.2]	0.67	-527.3 [-4411.3, 3356.8]	0.79
	≥60 y/o	725	50572	Ref.	-	Ref.	

* simple linear model, ** Linear model adjusted for Vaccinated and age groups.

Table S4. Determinants of antibody magnitude among the vaccinated group including number of dose.

		Multivariate Analysis	
Covariate	Category	Model estimate *	P-value
Age groups	2-11 y/o	-14970.0 [-30787.4, 847.5]	0.064
	12-17 y/o	-12906.0 [-28035.1, 2223.1]	0.095
	18-29 y/o	-1585.1 [-6121.2, 2951.1]	0.494
	30-59 y/o	-2960.9 [-7142.6, 1220.9]	0.165
	≥60 y/o	Ref.	-
Sex	Male	Ref.	-
	Female	2705.7 [-169.4, 5580.9]	0.065
Type of vaccine	Astrazeneca	Ref.	-
	Abdala	**24325.6 [9358.6, 39292.6]**	**0.002**
	Pfizer	**13753.9 [8379.3, 19128.6]**	**<0.001**
	Soberana	**35238.5 [19073.8, 51403.2]**	**<0.001**
	Sputnik	2473.7 [-1229.7, 6177.1]	0.191
Vaccination status	Incomplete	Ref.	-
	Complete	**7181.5 [4122.8, 10240.2]**	**<0.001**
	Homotypic boost	2105.7 [-2537.3, 6748.6]	0.374
	Heterotypic boost	956.8 [-10600.0, 12513.5]	0.871
Days after last dose	Titer change by day	-0.5 [-1.4, 0.3]	0.222
	1	Ref.	-
Number of dose	2	**5872.1 [2806.2, 8938]**	**<0.001**
	3	**5361.4 [1739.4, 8983.3]**	**0.004**
	Yes	Ref.	-
Self-reported SARS-CoV-2 infection	Unsure	-5866.3 [-14256.5, 2524.0]	0.171
	No	-2270.6 [-6609, 2067.8]	0.305

*Linear model adjusted by age groups, sex, type of vaccine, vaccine status, days after last dose and self-reported SARS-CoV-2 infection.

Table S5. Stratified antibody dynamics by vaccine type and vaccine status.

Type of Vaccine	Vaccination Status	*n*	Days after Last Dose - Median (IQR)	Titer Change by Day *	P-Value	Adjsted p-Value **
ASTRAZENECA	Incomplete	351	173 (102-309)	-27.0 [-53.6, -0.3]	0.047	0.206
	Complete	189	98 (62-226)	-16.7 [-60.7, 27.4]	0.457	0.697
	Homotypic boost	113	83 (60-161)	**79.3 [7.9, 150.7]**	**0.030**	0.157
	Heterotypic boost		64 (33-240)	9.7 [-111.4, 130.7]	0.869	0.904
SPUTNIK	Incomplete	123	248 (205-323)	**57.2 [5.6, 108.8]**	**0.030**	0.157
	Complete	322	124.5 (93-228)	**51.2 [15.8, 86.6]**	**0.005**	0.116
	Homotypic boost	158	55.5 (31-179)	43.0 [-7.6, 93.5]	0.095	0.347
	Heterotypic boost	1	-	-	-	-
SOBERANA	Incomplete	168	82 (46-166)	-39.8 [-99.2, 19.5]	0.187	0.458
	Complete	36	51 (35-177)	-93.2 [-246.7, 60.3]	0.225	0.458
ABDALA	Incomplete	526	97 (55-178)	-24.7 [-54.6, 5.3]	0.107	0.347
	Complete	141	55 (33-139)	**63.2 [8.2, 118.1]**	**0.025**	0.157
PFIZER	Incomplete	114	117 (91-209)	-15.8 [-71.2, 39.6]	0.573	0.745
	Complete	113	83 (50-161)	-19.2 [-79.0, 40.6]	0.526	0.720

* Linear models adjusted by age, ** Benjamini-Hochberg p-value correction for multiple testing.

Table S6. Determinants of antibody magnitude among the vaccinated with time interactions.

Covariate	Category	n	Model estimate (Magnitude) *	P-value	Model estimate (Dynamics titer/day) **	P-value
Age groups	2-11 y/o	420	-15457.4 [-32849.3, 1934.5]	0.082	-	-
	12-17 y/o	457	-14663.7 [-31333.5, 2006.2]	0.085	-	-
	18-29 y/o	456	1425.3 [-3545.0, 6395.6]	0.574	-	-
	30-59 y/o	509	-2418.7 [-6896.1, 2058.6]	0.290	-	-
	>=60 y/o	512	Ref.	-	-	-
Sex	Male	829	Ref.	-	-	-
	Female	1525	2214.3 [-897.5, 5326.0]	0.163	-	-
Type of vaccine	Astrazeneca	653	Ref.	-	Ref.	
	Abdala	667	**28572.3 [11317.7, 45826.9]**	**0.0012**	-7.0 [-40.1, 26.0]	0.677
	Pfizer	227	**15516.7 [6272.4, 24760.9]**	**0.001**	-22.0 [-68.9, 24.8]	0.357
	Soberana	204	**40680.4 [21358.5, 60002.2]**	**<0.001**	-39.2 [-95.5, 17.2]	0.173
	Sputnik	603	**-8845.3 [-15955.4, -1735.2]**	**0.015**	**40.3 [8.4, 72.3]**	**0.014**
Vaccination Status	Incomplete	1282	Ref.	-	Ref.	
	Complete	801	**6289.2 [629.3, 11949.1]**	**0.030**	10.9 [-17.2, 39.1]	0.447
	Homotypic boost	271	-190.7 [-8827.0, 8445.6]	0.966	32.9 [-15.1, 80.9]	0.179
Days after last dose		2354	-	-	-	-
	Titer change by day	-	-6.4 [-28.7, 16.0]	0.576	-	-
Self-reported COVID	Yes	248	Ref.	-	-	-
	Unsure	76	-2382.2 [-11552.5, 6788.1]	0.611	-	-
	No	2030	-1357.9 [-6164.7, 3448.8]	0.580	-	-

* Multivariate linear regression adjusted by age groups, sex, type of vaccine, vaccine status, days after last dose and self-reported SARS-CoV-2 infection and the interaction of vaccination status and type with days after last dose. Estimates show the main effects of age, sex, type of vaccine, vaccination status, days after last dose and self-reported covid. ** Interaction terms between time since last dose and type of vaccine and vaccination status in the same multivariate model
